# CRAFITY and AFP/PIVKA-II Kinetics Predict Prognosis in Hepatocellular Carcinoma on Immunotherapy

**DOI:** 10.3390/cancers17183058

**Published:** 2025-09-18

**Authors:** Shou-Wu Lee, Yi-Jie Huang, Ying-Cheng Lin, Hsin-Ju Tsai, Chia-Chang Chen, Chung-Hsin Chang, Teng-Yu Lee, Yen-Chun Peng

**Affiliations:** 1 Division of Gastroenterology, Department of Internal Medicine, Taichung Veterans General Hospital, Taichung 407219, Taiwan; 2 Department of Internal Medicine, Chung Shan Medical University, Taichung 40201, Taiwan; 3 Department of Post-Baccalaureate Medicine, College of Medicine, National Chung Hsing University, Taichung 402, Taiwan; 4 Department of Internal Medicine, National Yang Ming Chiao Tung University, Taipei 30010, Taiwan

**Keywords:** AFP, CRP, hepatocellular carcinoma, PIVKA-II

## Abstract

Background: The CRAFITY score, integrating baseline C-reactive protein (CRP) and alpha-fetoprotein (AFP), has been validated as a prognostic biomarker in hepatocellular carcinoma (HCC) treated with immunotherapy, but many patients present with non-elevated AFP, limiting its accuracy. This study evaluated a composite model incorporating the CRAFITY score with AFP/PIVKA-II kinetic changes. Methods: We retrospectively enrolled 69 patients with unresectable HCC (BCLC stage B/C) receiving immunotherapy between September 2021 and June 2023. Baseline CRP, AFP, and PIVKA-II, as well as 4-week changes, were recorded. The CRAFITY-100 RULE combined CRAFITY (0–2) with AFP/PIVKA-II kinetics (0–3), yielding three risk levels (I–III). Clinical outcomes included objective response (OR) and overall survival (OS). Results: Of the cohort, 10 (14.5%), 29 (42%), and 30 (43.5%) patients had CRAFITY scores 0, 1, and 2, respectively, but this score did not clearly stratify OS (median 24, 12, and 15 months; *p* = 0.267). In contrast, the CRAFITY-100 RULE classified 5 (7.3%), 35 (50.7%), and 29 (42%) patients into levels I–III, respectively, with significantly different survival (median OS 24, 15, and 7 months; *p* = 0.048). OR rates were lowest at level III (17%). Time-dependent ROC analysis confirmed superior discrimination of CRAFITY-100 RULE over CRAFITY scores at 6 months (AUROC 0.673 vs. 0.604) and 12 months (0.732 vs. 0.656). Conclusions: The CRAFITY-100 RULE provided clearer stratification and higher discrimination. This simple model integrating baseline and dynamic biomarkers may assist clinical decision-making in unresectable HCC treated with immunotherapy.

## Simple Summary

Patients with unresectable hepatocellular carcinoma (HCC) often receive immune checkpoint inhibitors, but early identification of responders remains challenging. The established CRAFITY score combines C-reactive protein and alpha-fetoprotein (AFP) at baseline, yet its prognostic value is limited, especially in AFP-negative disease. We developed the CRAFITY-100 RULE by integrating baseline AFP and PIVKA-II thresholds with their early (4-week) kinetic changes. In a real-world Taiwanese cohort of 69 patients, the new score provided clearer survival stratification and superior discrimination compared with the original CRAFITY. Time-dependent ROC analysis further showed higher AUROC values for CRAFITY-100 RULE at 6 and 12 months. This simple tool, based on routine and dynamic biomarkers, may assist clinicians predicting treatment outcomes and guide timely therapeutic decisions for patients receiving immunotherapy.

## 1. Introduction

Hepatocellular carcinoma (HCC) is the most common primary malignancy of the liver and a leading cause of cancer-related mortality worldwide [1]. Despite the advances in surveillance and early detection, a large proportion of patients are diagnosed at intermediate to advanced stages, where curative interventions such as surgical resection, transplantation, or local ablative therapies are not feasible [2]. In these cases, systemic therapies, including targeted therapies and immune checkpoint inhibitors (ICIs), play a crucial role in disease management.

ICIs, especially the combination of anti-PD-L1 and anti-VEGF agents (e.g., atezolizumab and bevacizumab) or anti-PD-L1 and anti-CTLA-4 (e.g., durvalumab and tremelimumab), have demonstrated superior efficacy compared to sorafenib in first-line treatment of unresectable HCC, as evidenced by the IMbrave150 [3] and HIMALAYA trial [4]. However, therapeutic responses vary widely among patients, with only a subset achieving objective responses or meaningful survival benefits. Consequently, there is a growing demand for reliable prognostic and predictive biomarkers to guide treatment decisions and stratify patient risk.

Alpha-fetoprotein (AFP), a serum glycoprotein produced by fetal liver cells and HCC cells, is the most commonly used biomarker in HCC. Its limitations include suboptimal sensitivity and specificity, as approximately 30–40% of HCC patients may present with normal AFP levels despite having advanced disease [5,6]. Another promising biomarker, protein induced by vitamin K absence or antagonist-II (PIVKA-II), has demonstrated superior specificity for HCC and has been incorporated into various prognostic models and surveillance algorithms [7,8,9]. Elevated PIVKA-II levels had been demonstrated to correlate with tumor aggressiveness [8], and its utility in monitoring therapeutic responses and predicting survival outcomes in HCC patients were also provided [10,11]. Several studies have suggested that dynamic changes in these biomarkers, particularly reductions after therapy initiation, may reflect tumor response and prognosis more accurately than baseline values alone [12,13,14,15,16].

In recent years, the CRAFITY score, which integrates baseline C-reactive protein (CRP) and AFP levels, has been proposed as a prognostic tool to predict survival in HCC patients undergoing immunotherapy [17]. Nevertheless, its accuracy is limited in patients with non-elevated AFP at baseline. Furthermore, dynamic changes in tumor markers, such as AFP and PIVKA-II, during treatment may reflect tumor response more sensitively than static baseline values. Therefore, incorporating kinetic changes in AFP and PIVKA-II into the prognostic model may provide a more comprehensive evaluation of treatment response and outcomes.

To address these limitations, we propose a novel composite scoring model that incorporates the CRAFITY score with the early kinetic changes in AFP and PIVKA-II, referred to as the CRAFITY-100 RULE score. This study aims to evaluate the prognostic significance of the CRAFITY-100 RULE score in patients with unresectable HCC receiving immunotherapy-based therapies.

## 2. Methods

### 2.1. Study Design and Patient Selection

This retrospective study enrolled patients with unresectable HCC (Barcelona Clinic Liver Cancer [BCLC] stage B or C) who received immunotherapy-based therapy as the first-line agent at Taichung Veterans General Hospital between September 2021 and June 2023. Inclusion criteria included age ≥ 20 years, histologically or radiologically confirmed HCC based on the AASLD criteria [18], received at least one cycle of immunotherapy, availability of serum AFP and PIVKA-II levels at baseline and 4 weeks post-treatment initiation, and adequate clinical follow-up and radiologic assessment. Patients were excluded if they had incomplete imaging or biomarker data, concurrent malignancies, prior liver transplantation, or follow-up duration less than 8 weeks. This study was conducted in accordance with the Declaration of Helsinki and approved by the ethics committees of Taichung Veterans General Hospital (CE23139B), and the requirement for written informed consent was waived.

### 2.2. Data Collection

Clinical data including age, sex, hepatitis B virus (HBV), hepatitis C virus (HCV), Albumin-Bilirubin (ALBI) grade, and tumor characteristics, including BCLC stage, MVI (macrovascular invasion) and EHS (extrahepatic metastasis) were extracted. Laboratory values of baseline CRP, AFP, PIVKA-II levels, and AFP, PIVKA-II levels at 4 weeks post-treatment initiation, were recorded. Radiological responses were assessed using modified RECIST criteria [19].

### 2.3. Scoring System: CRAFITY and 100 RULE

Baseline thresholds of AFP ≥ 100 ng/mL and PIVKA-II ≥ 100 mAU/mL were chosen in accordance with prior literature and clinical guidelines [15,20]. Early biochemical response was defined as ≥10% decline at 4 weeks, supported by previous reports linking AFP kinetics with treatment outcomes in systemic and immunotherapy settings [20,21,22].

**Figure 1:**
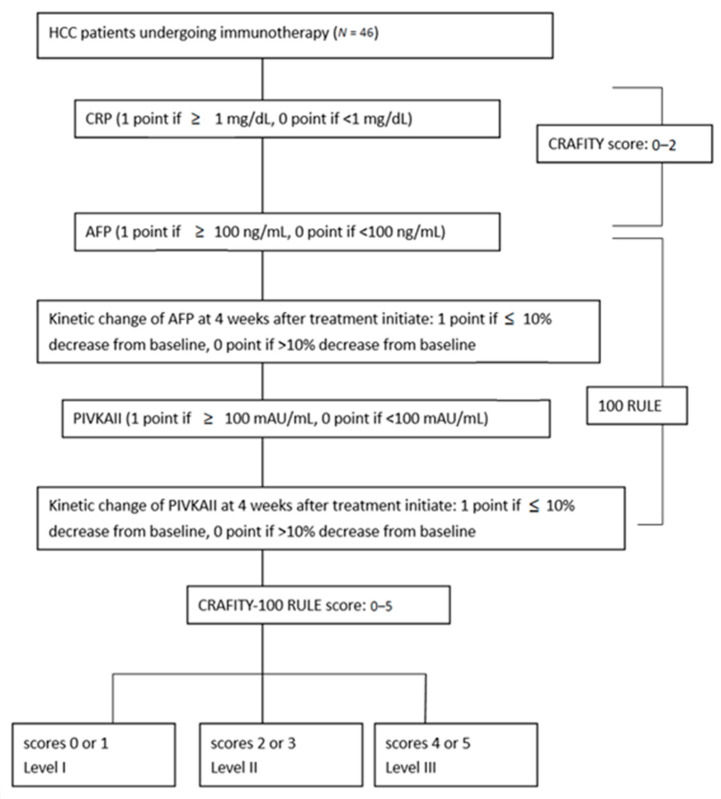
Illustrates the patient selection flow. Abbreviations: AFP, alpha-fetoprotein; CRP, C-reactive protein; HCC, hepatocellular carcinoma.

Figure 1 illustrates the patient scoring process. The CRAFITY score was calculated as follows: CRP ≥ 1.0 mg/dL = 1 point; AFP ≥ 100 ng/mL = 1 point. Total score ranged from 0 to 2. To assess early biomarker kinetics, the 100 RULE was devised: baseline PIVKA-II ≥ 100 mAU/mL = 1 point; AFP reduction < 10% at 4 weeks = 1 point; PIVKA-II reduction < 10% at 4 weeks = 1 point. The CRAFITY-100 RULE score combines the CRAFITY score (0–2) and 100 RULE (0–3), giving a total possible score of 0–5. Patients were stratified into three levels: level I = score 0–1, level II = score 2–3, level III = score 4–5. To facilitate application and enhance intuitive understanding, a checklist version is also provided in Table S1.

### 2.4. Endpoints

The primary outcomes were radiological objective response (OR), defined as complete or partial response per modified RECIST criteria, and overall survival (OS), defined as time from treatment initiation to death or last follow-up.

### 2.5. Statistical Analysis

Categorical variables were presented as frequencies and percentages, compared using Pearson’s chi-square test. Continuous variables were presented as means with standard deviations (SD) and compared using independent *t*-tests. Univariate and multivariate Cox regression models identified prognostic factors for OS. Kaplan–Meier method and log-rank test were used for survival analysis. A *p*-value < 0.05 was considered statistically significant. In addition, exploratory time-dependent AUROC (Area Under the Receiver Operating Characteristic Curve) values analyses were conducted to compare the discriminative ability of the CRAFITY and CRAFITY-100 RULE levels for overall survival at 6 and 12 months. To address sample size limitations, we conducted repeated internal 10-fold cross-validation for discrimination and calibration.

## 3. Results

### 3.1. Patient Characteristics and Radiological Response

The baseline characteristics are summarized as Table 1. A total of 69 patients with unresectable HCC met the inclusion criteria, and the ICI regimens included atezolizumab plus bevacizumab (*n* = 34), nivolumab (*n* = 23), or pembrolizumab (*n* = 12). The median age was 64.9 years, and 79.7% of cases were male. The etiology of liver disease was HBV (53.6%) and HCV (31.9%). Most patients had BCLC stage C (85.5%), and the distribution of ALBI grade 1 and 2/3 were 55.1% and 44.9%, respectively. About 62.3% of cases had MVI and 40.6% of cases had EHS. The best radiological response was shown in Table 2. There were 20 patients (29%) with OR, and 49 patients (71%) with non-OR to immunotherapy.

### 3.2. Distribution of CRAFITY Scores and CRAFITY-100 RULE

The results of CRAFITY scores and CRAFITY-100 RULE were listed in Table 3. The levels of baseline parameters, including AFP ≥ 100 ng/mL, PIVKA-II ≥ 100 mAU/mL and CRP ≥ 1 mg/dL, were similar between the patients with OR and those with non-OR. On the contrary, patients with OR had significant lower ratio of biomarkers decline < 10% from baseline at 4 weeks after immunotherapy started (AFP, 40% vs. 65.3%, *p* = 0.049; PIVKA-II, 30% vs. 73.5%, *p* = 0.001). There were 10 (14.5%), 29 (42%) and 30 (43.5%) patients classified as CRAFITY score 0, 1 and 2, respectively, but no significant difference existed between the subgroup of OR and the subgroup of non-OR (*p* = 0.209). 5 (7.3%), 35 (50.7%) and 29 (42%) patients were categorized as the CRAFITY-100 RULE level I, level II and level III, respectively. The distribution of CRAFITY-100 RULE levels was significantly different in these two subgroups (*p* = 0.015). A significant lower portion of level III patients (5/29, 17%) achieved an OR.

### 3.3. Overall Survival

As shown in Table 4, patients’ OS and the associated clinical factors were analyzed. Univariate Cox regression model showed ALBI grade 1 (HR 0.38, 95% CI 0.18–0.83, *p* = 0.015) and negative of MVI (HR 0.31, 95% CI 0.12–0.78, *p* = 0.013) had associated with better OS, and baseline PIVKAII ≥ 100 mAU/mL (HR 4.42, 95% CI 1.33–14.69, *p* = 0.015) and CRP ≥ 1 mg/dL (HR 2.85, 95% CI 1.06–7.65, *p* = 0.038) had poor OS. However, these clinical factors all lose their significant impact to OS after analysis per multivariate Cox regression.

**Figure 2:**
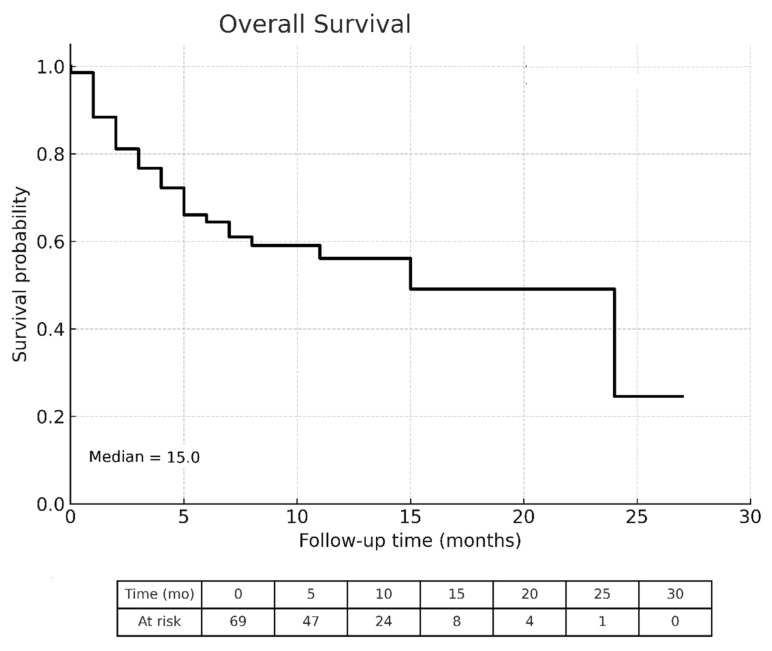
Kaplan–Meier analysis of overall survival in all patients.

**Figure 3:**
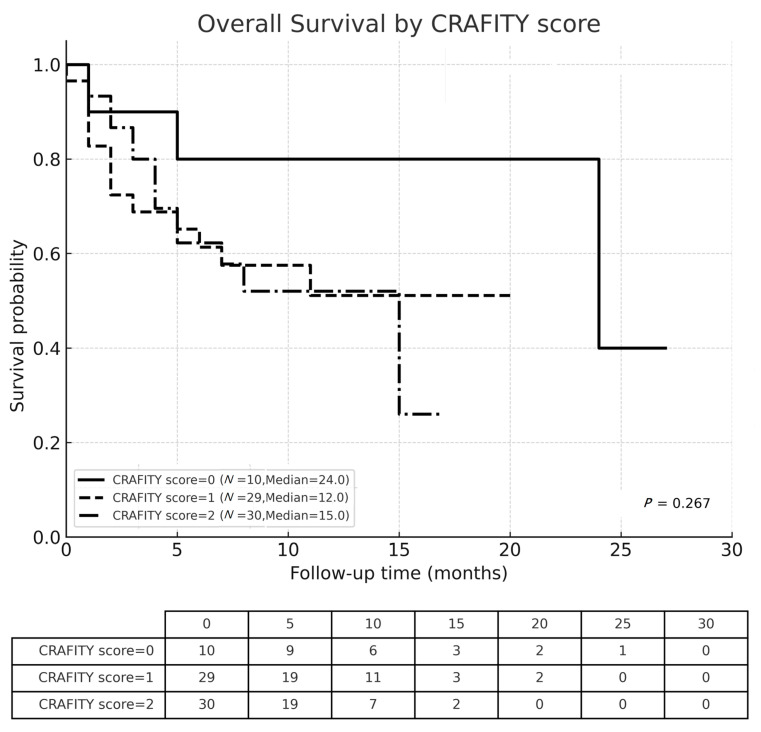
Kaplan–Meier analysis of overall survival in patients classified by the CRAFITY score.

**Figure 4:**
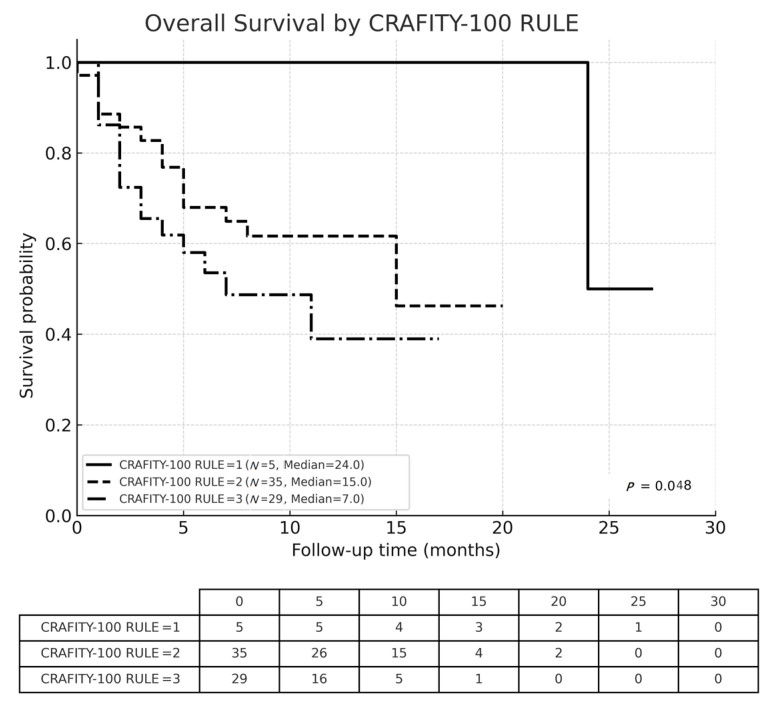
Kaplan–Meier analysis of overall survival in patients classified by the CRAFITY-100 RULE. (level I: score 0–1, level II: score 2–3, level III: score 4–5).

The OS of all patients, classified by CRAFITY score and CRAFITY-100 RULE were displayed in Figure 2, Figure 3 and Figure 4, respectively. The median OS of all individuals was 15 months. The Kaplan–Meier curves demonstrated significant different survival based on CRAFITY-100 RULE levels, with median OS of level I, II III were 24, 15 and 7 months, respectively (*p* = 0.048). On the contrary, the Kaplan–Meier curves did not demonstrate clear separation of survival based on CRAFITY scores (median OS of score 0, 1, 2 was 24, 12, 15 months, respectively; *p* = 0.267).

Exploratory time-dependent ROC analyses were performed to compare the discriminative ability of the original CRAFITY scores and CRAFITY-100 RULE (Figures S1 and S2). At 6 months, the AUROC value was 0.604 for the CRAFITY scores and 0.673 for the CRAFITY-100 RULE. At 12 months, AUROC values were 0.656 and 0.732, respectively. Further internal validation was performed using repeated 10-fold cross-validation comparing the discriminative performance of the CRAFITY scores and CRAFITY-100 RULE. Metrics include Harrell’s C-index and time-dependent AUROC at 6 and 12 months. The CRAFITY-100 RULE consistently achieved higher discrimination than the original CRAFITY scores across all measures (Table S2).

## 4. Discussion

Our study proposes the CRAFITY-100 RULE, which integrates baseline inflammatory and tumor markers (CRP, AFP, PIVKA-II) with their early kinetic changes, to better stratify patients with unresectable HCC undergoing immunotherapy. The unique value of this model lies in its ability to incorporate both static and dynamic biomarker information. While the original CRAFITY score did not significantly separate OS in our cohort, the CRAFITY-100 RULE demonstrated clear stratification. The incremental value was further supported by time-dependent ROC analyses, where the CRAFITY-100 RULE outperformed the CRAFITY score at both 6 and 12 months.

The CRAFITY score alone has been shown to correlate with OS in HCC patients treated with ICIs, as demonstrated by Scheiner et al. [13] and subsequently validated in external cohorts [23,24,25]. Its prognostic utility has been confirmed in patients receiving ICIs combined with transarterial chemoembolization (TACE) [25] or tyrosine kinase inhibitors (TKIs) [24]. This is important, as TKIs are still considered integral components of treatment strategies for patients with unresectable HCC, both in the first-line and second-line settings [26,27]. However, its utility remains limited in AFP-negative HCC. In our cohort, approximately 41% of HCC patients had baseline AFP < 100 ng/mL, a population often overlooked by AFP-based models.

Dynamic biomarker changes have been increasingly recognized as early surrogate markers of immunotherapy response. Several studies have reported that early AFP declines predict favorable outcomes in HCC patients receiving ICI-based treatments [12,13,14,15,16]. Similarly, PIVKA-II dynamics have shown utility in prognostication, particularly in Asian populations [28].

By incorporating both AFP and PIVKA-II kinetics into a unified scoring model, our study builds upon and significantly improves the prognostic capabilities of the CRAFITY score. The strong correlation between lower scores (level I) and both higher radiologic OR and prolonged survival reinforces the clinical validity of this approach.

The CRAFITY-100 RULE offers several practical advantages, as it is easily applicable using routine blood tests, incorporates early treatment kinetics to guide continuation or switching of therapies, identifies poor responders early (level III), or prompting consideration of alternative strategies, such as dual ICI or clinical trials. This scoring system may guide risk stratification in real-world treatment settings.

This study has several limitations. First, the modest cohort size (*n* = 69) and the very small number of patients in level I (*n* = 5) limit the stability of subgroup estimates. We therefore performed internal cross-validation analyses, and the results supported the results of our primary findings. A further multi-center project aimed at enrolling more patients to provide the external validation has been scheduled. Second, the heterogeneity of regimens may confound regimen-specific effects, though our score was designed to be regimen-agnostic. Third, although the scoring system may appear complex, it operationalizes only five binary checks using routine tests, mapped to three strata. This design makes the tool simple and feasible for clinical practice. Fourth, biomarker kinetics were available only at 4 weeks, and future prospective studies should incorporate serial biomarker monitoring to refine the dynamic component of the score. Fifth, collinearity is a concern when including both baseline and kinetic versions of the same biomarker in Cox models. We therefore emphasize parsimonious model specifications and highlight that the CRAFITY-100 RULE itself effectively summarizes overlapping information. Finally, comorbidities and concomitant medications were not systematically collected. Prior studies indicate that metabolic comorbidities [29] and medications such as angiotensin receptor blockers [30] may influence prognosis. These factors should be incorporated into future external validation. However, to our knowledge, this is the first study to refine the CRAFITY score by incorporating AFP and PIVKA-II kinetics, thereby improving prognostic discrimination in an Asian real-world cohort.

## 5. Conclusions

The CRAFITY-100 RULE, integrating CRP, AFP, and PIVKA-II levels at baseline and their early kinetic changes, represents a powerful prognostic tool in patients with unresectable HCC receiving immunotherapy. This model enables early identification of patients likely to respond favorably to immunotherapy, as well as those at high risk of poor outcomes.

## Figures and Tables

**Table 1:** Baseline characteristics in patients at the initiation of immunotherapy.

Variables Mean ± SD or *N* (%)		Total *N* = 69 (100%)	OR *N* = 20 (29.0%)	Non-OR *N* = 49 (71.0%)	*p*-Value
Age (years)		64.9 ± 11.6	61.2 ± 9.5	66.5 ± 12.1	0.083
	>65	38 (55.1%)	7 (35%)	31 (63.3%)	0.032
Gender	male	55 (79.7%)	16 (80.0%)	39 (79.6%)	0.624
	female	14 (20.3%)	4 (20.0%)	10 (20.4%)	
Viral hepatitis	HBV	37 (53.6%)	9 (45.0%)	28 (57.1%)	0.359
	HCV	22 (31.9%)	10 (50.0%)	12 (24.5%)	0.039
ALBI grade	1	38 (55.1%)	13 (65.0%)	25 (51.0%)	0.290
	2/3	31 (44.9%)	7 (35.0%)	24 (49.0%)	
BCLC stage	B	10 (14.5%)	5 (25.0%)	5 (10.2%)	0.113
	C	59 (85.5%)	15 (75.0%)	44 (89.8%)	
MVI		43 (62.3%)	11 (55.0%)	32 (65.3%)	0.423
EHS		28 (40.6%)	6 (30.0%)	22 (44.9%)	0.253
Concurrent LRT		23 (33.3%)	8 (40.0%)	15 (30.6%)	0.453
AFP (ng/mL)		21,072.2 ± 48,053.5	22,042.7 ± 44,476.8	20,667.9 ± 49,914.8	0.915
PIVKA-II (mAU/mL)		4429.2 ± 8864.9	3798.1 ± 9636.5	4692.1 ± 8616.8	0.708
CRP (mg/dL)		3.2 ± 3.1	1.9 ± 1.8	3.7 ± 3.3	0.021

Abbreviations: AFP, alpha-fetoprotein; ALBI: Albumin-Bilirubin; CRP, C-reactive protein; EHS, extrahepatic spread; HBV, hepatitis B; HCV, hepatitis C; LRT, locoregional therapy; MVI, macrovascular invasion; *N*, number of patients; OR, objective response; SD, standard deviation.

**Table 2:** The best radiological tumor responses of patients receiving immunotherapy.

mRECIST	All (*N* = 69)	
	*N*	%
Complete response	4	(5.8%)
Partial response	16	(23.2%)
Stable disease	21	(30.4%)
Progressive disease	28	(40.6%)
Objective response (OR)	20	(29.0%)
Non-objective response (non-OR)	49	(71.0%)

Abbreviations: *N*, number of patients.

**Table 3:** Distribution of CRAFITY scores and CRAFITY-100 RULE.

Variables *N* (%)		Total *N* = 69 (100%)	OR *N* = 20 (29.0%)	Non-OR *N* = 49 (71.0%)	*p*-Value
Baseline					
AFP ≥ 100 ng/ml		41 (59.4%)	14 (70.0%)	27 (55.1%)	0.253
PIVKA-II ≥ 100 mAU/mL		51 (73.9%)	13 (65.0%)	38 (77.6%)	0.281
CRP ≥ 1 mg/dL		48 (69.6%)	10 (50.0%)	38 (77.6%)	0.084
**At 4 weeks after immunotherapy**					
AFP decline < 10%		40 (58.0%)	8 (40.0%)	32 (65.3%)	0.049
PIVKA-II decline < 10%		42 (60.9%)	6 (30.0%)	36 (73.5%)	0.001
CRAFITY scores	0	10 (14.5%)	5 (25.0%)	5 (10.2%)	0.209
	1	29 (42.0%)	6 (30.0%)	23 (46.9%)	
	2	30 (43.5%)	9 (45.0%)	21 (42.9%)	
CRAFITY-100 RULE	level I	5 (7.3%)	4 (20.0%)	1 (2.0%)	0.015
	level II	35 (50.7%)	11 (55.0%)	24 (49.0%)	
	level III	29 (42.0%)	5 (25.0%)	24 (49.0%)	

Abbreviations: AFP, alpha-fetoprotein; CRP, C-reactive protein; N, number of patients; OR, objective response. CRAFITY-100 RULE: level I (score 0–1), level II (score 2–3), level III (score 4–5).

**Table 4:** The strength of association between clinical parameters and overall survival following immunotherapy usage.

	Univariate Analysis			Multivariate Analysis		
	HR	(95% CI)	*p*-Value	HR	(95% CI)	*p*-Value
Age (≤65 vs. >65 y/o)	0.77	(0.36–1.64)	0.502			
Gender (male vs. female)	1.03	(0.42–2.56)	0.945			
HBV (HBsAg + vs. −)	0.84	(0.40–1.77)	0.643			
HCV (anti-HCV + vs. −)	1.35	(0.60–3.08)	0.462			
ALBI grade (1 vs. 2/3)	0.38	(0.18–0.83)	0.015	0.54	(0.24–1.21)	0.138
BCLC stage (B vs. C)	0.29	(0.07–1.22)	0.091			
MVI (no vs. yes)	0.31	(0.12–0.78)	0.013	0.56	(0.21−1.52)	0.253
EHS (no vs. yes)	0.90	(0.43–1.93)	0.798			
Combined LRT (yes vs. no)	0.44	(0.18–1.10)	0.079			
Baseline						
AFP (≥100 vs. <100 ng/mL)	1.01	(0.47–2.14)	0.993			
PIVKAII (≥ 100 vs. <100 mAU/mL)	4.42	(1.33–14.69)	0.015	3.22	(0.93−11.11)	0.064
CRP (≥ 1 vs. <1 mg/dL)	2.85	(1.06–7.65)	0.038	2.14	(0.78−5.86)	0.137
At 4 weeks after immunotherapy						
AFP decline < 10% (yes vs. no)	1.87	(0.86–4.03)	0.112			
PIVKA-II decline < 10% (yes vs. no)	1.21	(0.55–2.62)	0.638			

Abbreviations: AFP, alpha-fetoprotein; ALBI: Albumin-Bilirubin; CI, confidence interval; CRP, C-reactive protein; EHS, extrahepatic spread; HBV, hepatitis B; HCV, hepatitis C; HR, hazard ratio; LRT, locoregional therapy; MVI, macrovascular invasion.

## Data Availability

The original contributions presented in this study are included in the article and Supplementary Materials. Further inquiries can be directed to the corresponding author.

## Supplementary Materials

The following supporting information can be downloaded at https://www.mdpi.com/article/10.3390/cancers17183058/s1: Table S1: Checklist format of the CRAFITY-100 RULE; Table S2: Internal validation using repeated 10-fold cross-validation; Figure S1: Time-dependent ROC curves comparing the prognostic performance of the CRAFITY score and the CRAFITY-100 RULE for overall survival at 6 months; Figure S2: Time-dependent ROC curves comparing the prognostic performance of the CRAFITY score and the CRAFITY-100 RULE for overall survival at 12 months.
