# Supplementary material for: CRAFITY and AFP/PIVKA-II Kinetics Predict Prognosis in Hepatocellular Carcinoma on Immunotherapy
Source: Cancers (Basel). 2025 Sep 18;17(18):3058. doi: 10.3390/cancers17183058 (PMC12468996; doi:10.3390/cancers17183058)
Supplement: Supplementary file 1 [file cancers-17-03058-s001.zip › cancers-3841302-supplementary.pdf]

Table S1. Checklist of the CRAFITY-100 RULE.

Component	Criteria	Points
Baseline AFP	≥ 100 ng/mL	1
Baseline PIVKA-II	≥ 100 mAU/mL	1
Baseline CRP	≥ 1.0 mg/dL	1
AFP kinetics at 4 weeks	<10% decline	1
PIVKA-II kinetics at 4 weeks	<10% decline	1
Total Score	0–5	

Risk stratification: Level I = Total score 0–1; Level II = Total score 2–3; Level III = Total score 4–5.

Table S2. Internal validation using repeated 10-fold cross-validation.

Model	C-index (mean ± SD)	AUROC at 6 months (mean ± SD)	AUROC at 12 months (mean ± SD)
CRAFITY score	0.551 ± 0.187	0.610 ± 0.203	0.678 ± 0.281
CRAFITY-100 RULE	0.622 ± 0.169	0.667 ± 0.181	0.737 ± 0.254

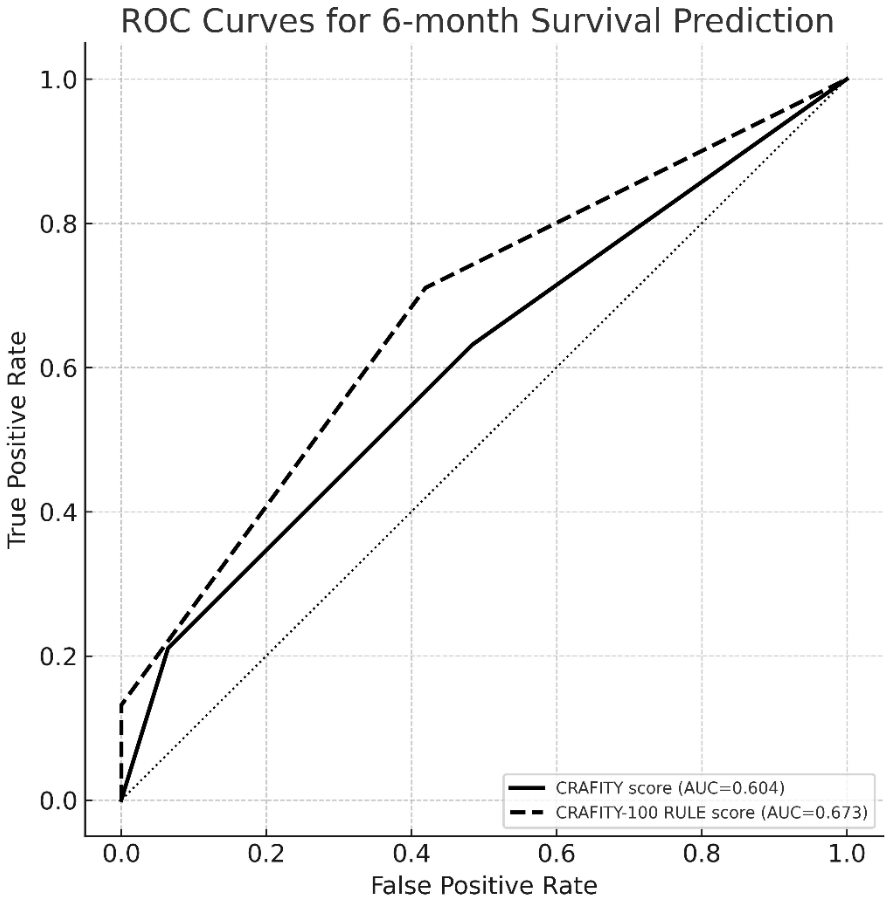

Figure S1. Time-dependent ROC curves comparing the prognostic performance of the CRAFITY score and the CRAFITY-100 RULE for overall survival at 6 months.

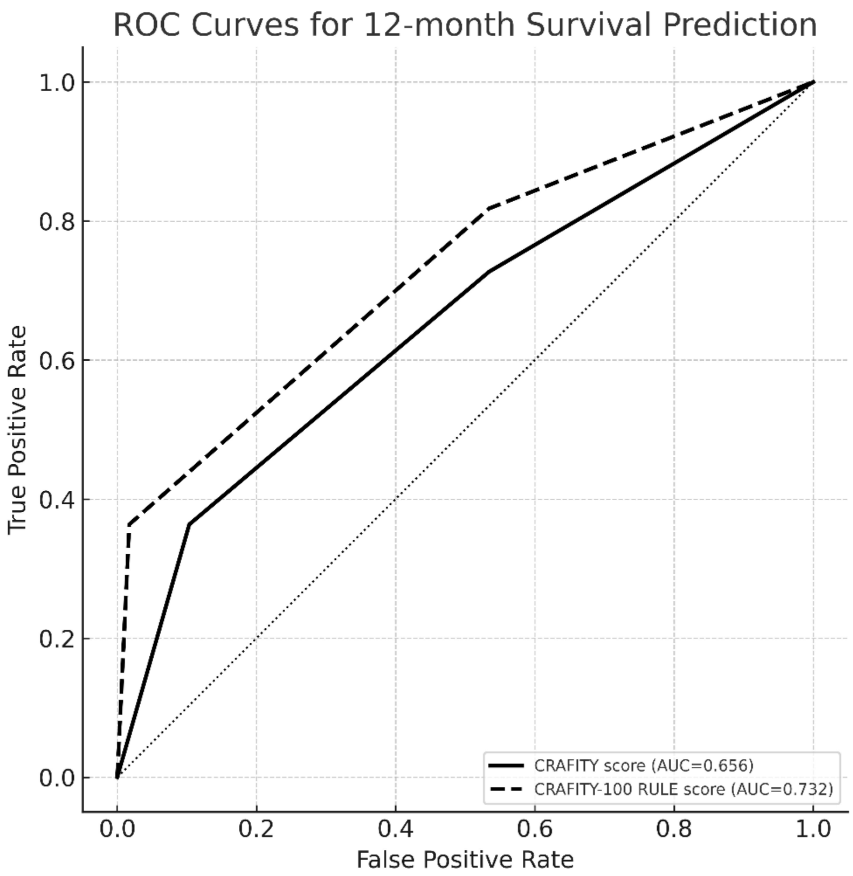

Figure S2. Time-dependent ROC curves comparing the prognostic performance of the CRAFITY score and the CRAFITY-100 RULE for overall survival at 12 months.
